# Nanoenabled Bioseparations: Current Developments and Future Prospects

**DOI:** 10.1155/2019/4983291

**Published:** 2019-01-29

**Authors:** Sherif Ashraf Fahmy, Mohamad Alawak, Jana Brüßler, Udo Bakowsky, Mayyada M. H. El Sayed

**Affiliations:** ^1^Department of Chemistry, American University in Cairo (AUC), AUC Avenue, P.O. Box 74, New Cairo 11835, Egypt; ^2^Department of Pharmaceutics and Biopharmaceutics, Philipps University of Marburg, Robert-Koch-Str. 4, 35037 Marburg, Germany

## Abstract

The use of nanomaterials in bioseparations has been recently introduced to overcome the drawbacks of the conventional methods. Different forms of nanomaterials, particularly magnetic nanoparticles (MNPs), carbon nanotubes (CNTs), casted nanoporous membranes, and electrospun nanofiber membranes were utilized in biological separation for the aim of production of different biomolecules such as proteins, amino acids, nucleic acids, and enzymes. This paper critically reviews the state-of-the-art efforts undertaken in this regard, with emphasis on the synthesis and performance evaluation of each nanoform. Challenges and future prospects in developing nanoenabled bioseparations are also discussed, for the purpose of highlighting potential advances in the synthesis and fabrication of novel nanomaterials as well as in the design of efficient nanoenabled processes for separating a wide spectrum of biomolecules.

## 1. Introduction

**Figure 1:**
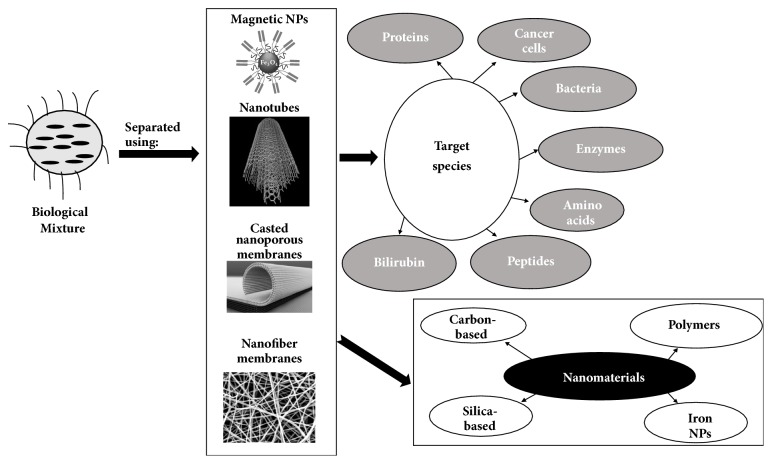
Different forms and types of nanomaterials used in bioseparation and their biological applications.

Nanotechnology is an emerging field of study which deals with matter at the nanolevel (1-100 nm). It has promising applications in many different fields such as drug delivery, diagnosis, industrial processing, and bioseparations [1]. The exceptional biological, physical, and chemical properties of nanomaterials are attributed to their high average surface area to volume ratio [2]. In the past few years, there has been a growing interest in utilizing nanotechnology in bioprocessing through the design of novel nanobiological objects (NBOs) that can be applied in bioseparation, imaging, and sensing of many different biological compounds [3]. Bioseparation can be defined as the effective isolation and purification of a certain biomolecule selectively from a complex biomixture. It plays a crucial role in different biological processes such as diagnosis, treatment, vaccination, and industrial production of biological compounds [4]. The most popular nanomaterials that have been utilized in bioseparation are carbon-based or silica-based inorganic materials, and polymeric materials, in addition to the iron oxide magnetic nanoparticles whose applications have recently emerged. These materials have been applied in various nanoforms including nanoparticles, nanotubes, and casted nanoporous and nanofiber membranes. An illustration of these nanomaterials, their forms, and their biological applications is depicted in Figure 1.

The conventional methods of bioseparations such as centrifugation, filtration, precipitation, and chromatography suffer from several drawbacks such as being time consuming, expensive, and of low throughput [5–7]. Consequently, there is an urgent need to develop novel, simple, cost effective, rapid, and high throughput methods as alternatives for the separation of biomolecules such as proteins, DNA, amino acids, enzymes, etc. [8].

Various studies addressed the use of nanomaterials in the separation of biomolecules. This paper critically reviews the state-of-the-art work that has been done in this area, with the aim of highlighting potential developments that could be undertaken in fabricating novel nanomaterials and/or designing effective methods and processes for separating different biomolecules. Emphasis will be given to studies that utilize bioseparation for producing biological compounds rather than for diagnostic or analytical purposes. These studies particularly employed zero-dimensional nanomaterials in the form of magnetic nanoparticles and one-dimensional nanomaterials in the form of carbon nanotubes, in addition to three-dimensional nanomaterials in the form of casted nanoporous membranes and electrospun nanofiber membranes. Thus, these nanoforms will be individually reviewed with regard to their synthesis, performance evaluation, and their applications in bioseparation.

## 2. Magnetic Nanoparticles (MNPs)

In the past few years, MNPs gained great attention in the field of bioseparations due to their numerous advantages which include, but are not limited to, (i) reduced agglomeration [9]; (ii) large surface area resulting from their tremendous surface to volume ratios [10, 11]; (iii) ability to perform all relevant separation steps in one single container [12]; (iv) ease of manipulation by external magnetic field which accelerates the separation process [11, 13, 14]; and most importantly (vii) their versatile particle size ranging from few up to tens of nanometers, which hence makes it suitable for separating a wide range of biomolecules such as proteins (5-50 nm) and cells (10-100 nm) [15]. However, the nonprotected or bare nanoparticles could be prone to oxidation [16, 17]. In bioseparations, iron oxide nanoparticles (Fe_3_O_4_ NPs) are the most commonly used NPs owing to their biocompatibility, nontoxicity, and the versatile well established methods by which they can be synthesized [9, 18]. This is in addition to their superparamagnetic properties where they show high magnetization in presence of an external magnetic field and zero magnetization in absence of this field thus minimizing aggregation, and this, in turn, gives them distinguished performance in bioseparation [9, 13, 19].

**Figure 2:**
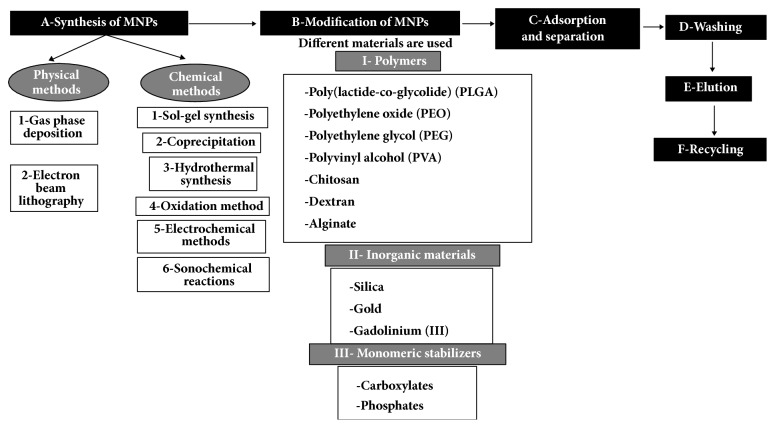
A schematic diagram summarizing the basic steps for bioseparation using MNPs.

Achieving a successful magnetic bioseparation takes place via six main steps (Figure 2) which can be presented as follows:

*(A) Synthesis of MNPs. *Many well-established physical and chemical methods are utilized for the preparation of MNPs such as electron beam lithography, sol-gel synthesis, coprecipitation, and sonochemical reactions. These methods are reviewed in detail elsewhere [11, 20, 21].

*(B) Modification and Functionalization of MNPs. *This step is crucial for increasing the stability of MNPs, decreasing their agglomeration in aqueous media, and enhancing biotargeting. Different materials were used for surface coating of MNPs such as polymers, inorganic materials, and monomeric stabilizers as presented in Figure 2. Silanization, using silane groups, is the most commonly used method for surface modification of MNPs due to the fact that it is relatively nonexpensive and of low toxicity and that it can be performed under various conditions and media. Furthermore, silane groups protect the core MNPs from potential oxidation [20, 21].

*(C) Adsorption and Separation. *In this step, functionalized MNPs bind to the desired biomolecules, present in the sample, via adsorption mechanisms, after which an external magnetic field is applied to retain the desired biomolecules together with the functionalized MNPs while the undesired ones are separated [20, 21].

*(D) Washing. *Herein, a washing buffer is used to collect the MNPs that are bound to the desired biomolecules. The magnetic field is switched on and off several times, where during the off-phase the MNPs are suspended, along with the unbound desired biomolecules, and during the on- phase the MNPs binding the target biomolecules are collected from the washing buffer.

*(E) Elution/Recovery. *An elution buffer is utilized in this step to recover the desired biomolecules.

*(F) Regeneration/Reuse. *This step is subsequent to separation, where the MNPs are incubated in fresh solutions to regenerate the binding sites on their surface such that they can be reused for further separations [21, 22].

To address the limitations of MNPs in terms of capacity and to develop their physicochemical properties, their surfaces were modified using biocompatible polymeric/organic or inorganic compounds. This proved advantageous in several aspects such as mitigating aggregation or sedimentation of the NPs in absence of external magnetic field, increasing their colloidal stabilities in solutions and facilitating the adsorption of target biomolecules on their surface and, hence, improving the selective separation of desired biomolecules from the solution, using magnetic field [9, 20]. In what follows, we will discuss how specific biomolecules such as proteins, DNA, and cells were separated using MNPs.

### 2.1. Separation of Proteins

**Figure 3:**
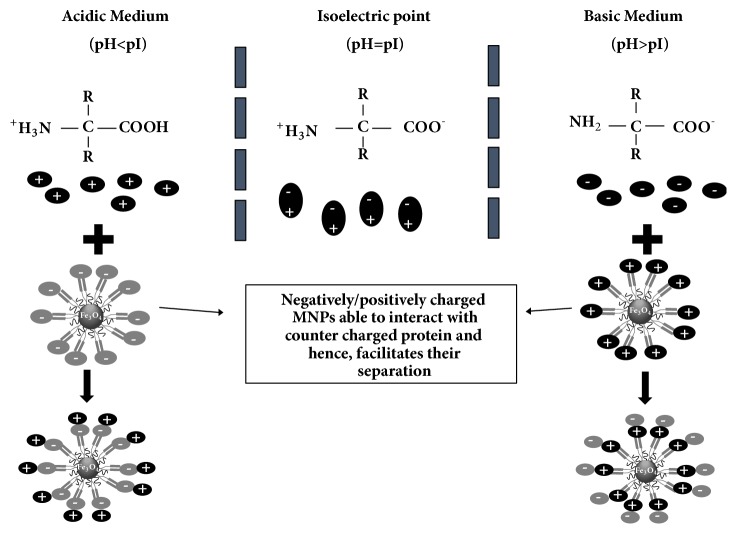
Schematic diagram for the electrostatic interaction between the amino acids and MNPs under different pH conditions.

Before reviewing the protein separation studies, we will give a brief overview of the protein structure and the main functional groups that interact during separation. Proteins are composed of twenty-two different amino acids which control the degree of their hydrophilicity, hydrophobicity, polarity, and nonpolarity. MNPs bind to different protein copolymers via several mechanisms such as ligand binding, van der Waals', hydrophobic, and electrostatic interactions [23–25]. The electrostatic properties of amino acids are the key for their bioseparation, since they exist as zwitterions, having equal positive and negative surface charges at their isoelectric point (pI), and their surface net charge can be manipulated by the pH of the surrounding media leading to either positively charged proteins (pH < pI) or negatively charged ones (pH > pI) (Figure 3). Consequently, charged proteins bind to their countercharged MNPs [26–28].

In previous work, a comparative study was held to investigate the binding of fetal bovine serum (FBS) protein to two differently charged Fe_3_O_4_ MNPs of average size 25-30 nm modified with negatively charged poly(acrylic acid) (PAA) and positively charged polyethyleneimine (PEI) functional groups [29]. Upon mixing FBS protein with the MNPs, their size increased by more than 300 nm due to electrostatic interactions, while their average surface charges decreased. In addition, the percent adsorption uptake capacities of human neuroblast SH-SY5Y cell (ATCC CRL-2266) culture onto PEI-MNPs and PAA-MNPs were found to be 54% and 27%, respectively, after 15 h, while their corresponding uptakes were 100% and 58% after 72 h. The higher uptake onto PEI-MNPs could be owed to its positive surface charge which favors the adsorption of the negatively charged proteins. This study showed the impact of surface chemistry on the adsorption of proteins and cells onto MNPs through electrostatic interactions [29]. In another study, lysozyme was effectively separated via adsorption mechanism using nonfunctionalized magnetite/silica core shell composite microspheres. The maximum binding capacity for lysozyme was found to be 127 mg/g. The magnetite/silica microspheres were functionalized with polyacrylic acid (PAA) chains resulting in increasing the binding capacity for lysozyme by 22 times in comparison to the nonfunctionalized magnetite/silica microspheres [30].

In addition to the electrostatic interactions, MNPs can bind to biomolecules via hydrophobic or affinity (coordination bonding) interactions. For instance, BSA was separated by hydrophobic partitioning realized by the functionalization of the silica coated MNPs with hydrophobic alkyl chains which, in turn, increased the number of hydrophobic sites that can bind BSA [31]. One advantage of hydrophobic MNPs is their ability to form clusters or “hydrophobic pockets” that capture proteins [31, 32]. Affinity MNPs, on the other hand, were also shown to be effective in separating proteins. Studies reported the design of Fe_3_O_4_ MNPs functionalized with carboxylic acid groups to separate 36 mg/g of purified trypsin from pancreatic extract using specific affinity ligand (soya bean trypsin inhibitor (SBTI)) attached to the functionalized MNPs [33]. Similar work also showed the successful separation of His-tag proteins by affinity MNPs. In one study, His-tag proteins were purified from* E. coli* lysate using a multifunctional magnetic mesoporous core/shell heteronanostructure formulation (Fe_3_O_4_/NiSiO_3_ core/shell nanostructure). The magnetite core was synthesized using a modified solvothermal method and was coated with SiO_2_ layer using ultrasonication. Afterwards, the hydrothermal method was undertaken to initiate the growth of the mesoporous nickel silicate shell via a chemical-template etching mechanism whereby the Fe_3_O_4_/NiSiO_3_ core/shell nanostructure was eventually formed. By virtue of the high affinity of nickel ions found on the surface of nanostructures, the separation of His-tagged proteins was achieved with high selectivity and a removal efficiency of up to 97.28%, 95.93%, 95.93%, and 88.42% for four operation cycles, using phosphate buffer saline at 4°C, while the captured protein was successfully recovered using imidazole [34]. Other workers were able to separate histidine tagged proteins from cell lysate using a chelate system of Co/Fe_2_O_3_-DA-NTA-Ni^2+^. In this system, cobalt was used to enhance the adsorption of dopamine (DA) onto Fe_3_O_4_ nanoparticles surface and increase their stability, while DA was used as a stable anchor to link the nitrilotriacetic acid (NTA) with the iron oxide MNPs. High salt concentration and 2% sodium dodecyl sulfonate (SDS) were used to increase the stability of the bond between DA and Fe_3_O_4_. For separation purposes, NTA functional group was chelated with immobilized Ni ions which bind to the protein histidine residues. Binding took place in presence of 20 mM Tris buffer (pH 7.9) and the MNP-protein system was captured by a small external magnet. Histidine was finally recovered via elution with imidazole [35]. In another recent study, his-tagged green fluorescent protein (GFP) was separated selectively using Fe_3_O_4_ nanoparticles coated with polyacrylamide (PAM) and functionalized covalently with NTA-Ni^2+^ (Fe_3_O_4_/PAM/NTA-Ni^2+^ MNPs). The average size, polydispersity index (PDI), and zeta potential of the final product were found to be 338.5 nm, 0.243, and -18.0, respectively, indicating good stability and dispersion for the MNPs. Results manifested the strong magnetic responsiveness of Fe_3_O_4_/PAM/NTA-Ni^2+^ MNPs which is crucial for rapid separation of proteins from solutions under the influence of external magnetic field, as well as their selective separation for his-tagged green fluorescent protein (GFP) which was demonstrated by their high adsorption efficiency (93.37%) [36].

A relatively recent development in the design of affinity MNPs is the incorporation of silica nanoparticles that facilitate bioconjugation. Streptavidin protein was separated by a core/multishell formulation comprising a core of silica NPs (200 nm), double-layered with Fe_3_O_4_ NPs (DL MNPs). An extra outer layer of silica coating was applied onto the DL MNPs to encapsulate them, increase biocompatibility, and facilitate functionalization. In order to separate streptavidin, DL MNPs were bioconjugated with biotin, through a typical amine functionalization onto the silica surface followed by amide coupling. The prepared NPs were highly uniform with minimal agglomeration and a size of 400 nm. They also possessed superparamagnetic properties with an ability to retain it for two months at room temperature. Although the particles demonstrated low magnetization in the magnetic field, large numbers of DL MNPs were attached to a single protein cell leading to a separation efficiency of streptavidin ranging from 70-90% higher than silica-coated Fe_3_O_4_ MNPs. Another important advantage for this formulation is its negligible cytotoxicity as detected by the CCK-8 assay [37].

### 2.2. Separation of DNA

The separation of DNA using MNPs is based on the same types of interactions encountered in proteins. As with proteins, the charge density distribution over the surface of DNA molecules controls their binding to the MNPs of counter charge [38, 39]. In physiological media, DNA molecules are negatively charged owing to their phosphate backbone. However, in acidic media, they become positively charged due to protonation of their phosphate groups [40]. One study availed of electrostatic interactions in order to separate salmon sperm DNA by means of magnetic mesoporous silica-magnetite nanocomposites prepared by the template-assisted method [41]. At the physiological pH (7.4) and high salt concentrations, the nanocomposites acquired a positive charge which, in turn, facilitated electrostatic interactions with the negatively charged phosphate backbones of DNA enabling their efficient separation. Approximately 100% of DNA was recovered from the surface of the nanocomposite, as opposed to less than 10% recovered from the magnetite core [41]. Using these nanocomposites, it was possible to separate the same amount of DNA that could be separated by the classical amorphous silica-magnetites but using half the quantity of materials [41–43]. In addition to electrostatic interactions, functionalization of the surfaces of MNPs with affinity ligands such as biotin and avidin enabled the separation of DNA molecules by virtue of affinity interactions (specific binding) between the MNPs and DNA [44].

### 2.3. Separation of Other Biomolecules

Owing to the versatility of functional groups that can be used to modify their surfaces, MNPs were employed in separating several biomolecules, other than proteins and DNA, such as cells, bacteria, genes, and viruses [45–47]. Anti-CD 3 monoclonal antibody bioconjugated to core/shell Fe_3_O_4_/Au MNPs, was successful in separating T-cells from the spleen with efficiency of up to 98.4 % [48]. MNPs with average sizes of 14-19 nm were prepared through the coprecipitation of Fe^+2^ and Fe^+3^ in water and, then, reduction with chloroauric acid (HAuCl_4_) which takes place on the surface of Fe_3_O_4_ MNPs forming a gold shell coating. Afterwards, the antibody was bioconjugated to the prepared Fe_3_O_4_/Au MNPs using protein A as an intermediate linker that is covalently immobilized on the surface of the MNPs, thus acting as an Fc receptor that captures the antibody's Fc domains [48].

The main aspects and findings of the above studies are summarized in Table 1.

## 3. Carbon Nanotubes (CNTs)

During the past twenty years, there has been a great interest in using CNTs for biotechnological applications [49]. This is because CNTs, which are composed of rolled sheets of carbon hexagons, have many advantages over other spherical nanoparticles which make them unique due to their large inner volumes and their accessible inner surfaces that allow for the incorporation of different desired biomolecules by virtue of their cylindrical structure. Their unique inner and outer surfaces also facilitate their functionalization by other groups or ligands, a property crucial for bioseparations. In addition, they have electric, thermal, and optical properties as well as exceptional mechanical strengths [49–51]. In this regard, many types of functionalized CNTs have emerged during the last few decades, examples of which are self-assembling CNTs, template-synthesized CNTs, fullerene CNTs, peptide CNTs, and organosilica polymeric CNTs [50, 52–55]. The template-synthesized method is considered as the most convenient for the preparation of functionalized CNTs since it provides a simple means for attaching chemical and biochemical groups (such as silica, gold antibodies, etc.) of different hydrophilicity and/or hydrophobicity on the inner and outer surfaces of CNTs and hence can be beneficial in separating various biomolecules [50]. For example, nanotubes with hydrophilic functional groups on their outer surfaces and hydrophobic groups on their inner surfaces can be used to extract lipophilic chemicals and drugs from aqueous solutions.

Few studies reported the use of CNTs in the separation of biomolecules. CNTs clusters have shown promising potential in separating different bacterial species, probably due to their high affinity to binding bacterial cells directly without the need for conjugating ligands or antibodies as well as their paramagnetic properties in presence of an external applied magnetic field [56, 57]. They were therefore utilized for the separation of various Gram (+) and Gram (-) bacteria [58]. This was accomplished using large clusters of multiwalled CNTs (MWCNTs) which were recommended over other types of CNTs such as single-walled CNTs and double-walled CNTs, particularly due to their relatively low cost, and ease of capturing high bacterial capacities [59]. Bacterial solutions were incubated with MWCNTs until all employed bacterial strains were spontaneously adsorbed onto the MWCNTs and, then, an external magnetic field was applied to separate the adsorbed bacteria. The adsorption capacities of all the targeted bacterial strains onto the MWCNTs were almost the same and were equal to 2 × 10^7^ CFU/L indicating that the bacterial cell surface properties have negligible effect on the adsorbing efficiency of MWNT clusters. The mechanisms by which the bacterial cells were adsorbed, nonselectively, to the MWCNTs are not yet known; however it could be owed to the hydrophobic interaction between the highly hydrophobic MWNT surface and the surface of bacteria. Consequently, MWCNTs acted as a universal adsorbent to almost most of the bacterial strains [58]. The ability of CNTs clusters to separate different types of bacteria can be beneficial to a variety of biological applications which require the removal of bacteria such as water purification, food processing, purification of implants, and the development of new generations of antimicrobial agents.

CNTs also showed promising results in the separation of proteins. Cup-stacked carbon nanotubes (CSCNTs) functionalized with high density of carboxyl groups and loaded with iron NPs were utilized to capture proteins. Functionalization with carboxylic acid groups was carried out by radical addition, followed by amidation using 3-aminophenylboronic acid (APBA) thus enabling it to interact with the antibody IgG through affinity interactions. After magnetic decantation, a separation capacity of 56 *μ*g/mg for the CSCNTs-APBA-IgG conjugate was achieved. The conjugate was also successful in capturing target antigens through antigen antibody reactions [60].

From the above, it is clear that limited work has been done on CNTs although there are myriad possibilities for their functionalization with specific groups that can potentially enhance their biotargeting properties.

## 4. Membranes

### 4.1. Casted Nanoporous Membranes

The use of mesoporous membranes (MPMs) in nanofiltration of biomolecules has dragged the attention of many researchers during the past few decades [61, 62]. To achieve efficient bioseparations, such membranes should be characterized by uniform controlled pore sizes of tens nm or less to ensure selective separation of the desired biomolecule, high chemical and physical stability, small membrane thickness to enhance the analyte flux, and, finally, well controlled surface charges [63–65].

MPMs have a wide variety of biological applications such as crystallization of proteins, separation of blood components, and synthesis of biosensors. More importantly, MPMs have a significant role in separating target proteins from a multicomponent system of biomolecules [66–69]. Separation of different-sized proteins and/or amino acids is based on size exclusion through controlled membrane pore sizes, in addition to surface interactions of the target proteins [70, 71]. Simply, when a biological mixture flows through the membrane, proteins with sizes larger than or equal to the molecular weight cutoff (MWCO) of the membrane will be retained leading to their biological separation [63–72]. However, if two or more proteins have comparable sizes and different charges, they can be separated using electrostatic interactions [73].

A number of studies demonstrated the use of electrostatic interactions for membrane separation of proteins as presented in Table 2. A couple of studies investigated the separation of bovine serum albumin (BSA) and bovine hemoglobin (BHb) from their mixture based on the difference in their isoelectric points (pI), since they both have comparable molecular weights of 66 and 65 kDa, respectively. In the first study, workers utilized gold-coated poly(carbonate) track-etched (PCTE) membranes synthesized by electroless gold deposition in order to form gold-coated nanopores within the PCTE membranes. Membranes were then immersed in an ethanolic solution of a weak acid alkane thiol to form the modified self-assembled monolayer (SAM-modified) gold nanotubular membranes (PCTE/Au/SAMs) which were used further in protein separation [74]. Both pH and ionic strength influenced separation. A selectivity of 67 was achieved at pH 4.7, with an average radius of 11 nm for the SAM-modified membrane nanopores. At this pH, BSA (pI = 4.7) was neutral, BHb (pI = 7.0) was positively charged, while the nanopores were negatively charged by virtue of the thiol groups bound to its surface. Consequently, BHb interacted electrostatically with the membrane while BSA had very weak interaction leading to a relatively high flux of the neutrally charged BSA and a low flux of the positively charged BHb [74–76]. In a second more recent study, the two proteins were successfully separated using thin anodic aluminum oxide membranes (AAO) of (0.7–1*μ*m) thickness and of narrow pore size distribution (20–30 nm diameter) and which were fabricated by anodizing aluminum films that were deposited on silicon substrates using the lithographic and etching method. Separation was achieved with high throughput (> 10^−8^ M.cm^−2^ s^−1^) and high selectivity (> 42) at pH 4.7, and with a transport rate of one order of magnitude higher than the conventional membranes [77]. The two aforementioned studies manifest the advantage of using electrostatic membrane interactions for separating proteins of comparable sizes and which could not be separated using size exclusion through the conventional nanofiltration membranes.

The concept of electrostatic interactions was also applied in separating proteins possessing different sizes. The separation of lysozyme/myoglobin binary mixture was achieved onto mesoporous silica membrane (SBA-15) with different pH buffer solutions. The process was based on manipulating the pH of the media in a way which favors the adsorption of only one of the two proteins, since they have two different isoelectric points of 11.35 and 7.3, respectively. At pH 10.6, exclusive binding of lysozyme took place with more than 85% of the entire lysozyme immobilized in the silica pores, whereas exclusive and complete binding of myoglobin took place at pH 4.5. However, the contrary of this behavior occurred at pH 3.8 where about 45% of the lysozyme adsorbed to the SBA-15, while myoglobin was fully displaced [78]. This is probably because both lysozyme and the protons in this low pH solution are competing with myoglobin over the adsorption sites. Recently, a group of workers developed ultra-thin crosslinked polymeric nanomembranes that were based on a thermoset resin constituting (poly[(o-cresyl glycidyl ether)-co-formaldehyde (PCGF), and poly(D,L-lactide-*co*-glycolide) (PLGA) cured with branched polyethyleneimine (PEI). The developed membranes possessed nanoscale perforations of 25 nm diameter and demonstrated low surface energies, high mechanical strength, and high efficiencies in separating proteins such as myoglobin, Cytochrome c, and BSA. Furthermore, they exhibited good biocompatibility, due to their hydrophilic nature, and resistance to high pHs and, hence, they were claimed to be suitable for application onto different biological fluids as single use devices [79].

In addition to proteins, MPMs were used to separate amino acids from multicomponent systems. Glycine was selectively separated from a mixture of four neutral amino acids: glycine, l-alanine, l-serine, l-glutamine, and one basic amino acid, l-lysine with different molecular weights of 75, 89, 105, 146, and 146 g mol ^−1^, and pIs of 6.10, 6.00, 5.70, 5.70, and 9.75, respectively. This was accomplished using a new class of nanofiltration (NF) membranes produced by depositing six to seven bilayers of poly(styrenesulfonate)/protonated poly(allylamine) hydrochloride (PSS/PAH) on porous alumina. The neutral amino acids were separated based on their size differences and the selectivity of glycine over l-glutamine was about 50 with a solution flux of 1.3 m^3^/(m^2^ day) [80]. The same separation principle was used by other workers to separate a mixture of l-glutamine/glutamate with 90% rejection of glutamate and nearly 85% permeation of l-glutamine to give an l-glutamine/glutamate selectivity of 21.7 along with a flux of 0.81 m^3^/(m^2^ day) [81].

Recently, the advance in microfluidic devices opened up opportunities for utilizing them in bioseparations. However, they were mainly used for diagnostic and detection purposes. One reported example was the separation of proteins from urine via a microfluidic device with two nanoporous PCTE membranes with different sized nanopores sandwiched between two polydimethylsiloxane (PDMS) pre-polymer layers with embedded channels. The principle of operation was based on size selective transport through membranes of different pore sizes. The first had a large pore size of 100 nm and it acted as a filter to screen out particles, cells and larger proteins, whereas the second had a small pore size of 10 nm that allowed the passage of small ions and molecules and retained larger ones. This microfluidic system provides an effective method for the quantitative diagnosis of albuminuria, an indicator of renal failure, and it overcomes the drawbacks of conventional tests. In addition, it offers a very fast and sensitive detection of HSA levels of above 30 *μ*g mL^−1^ in urine with a linear range of 0−100 *μ*g mL^−1^, a LOD of 1.5 *μ*g mL^−1^, and a recovery of HSA from urine of 81.2−116.8% [82]. As for the potential of using these microfluidic devices for production purposes, it is yet to be explored.

### 4.2. Nanofiber Membranes

Selective chromatographic adsorption is one of the traditional methods for industrial separation of biological molecules. It involves the adsorption of the target biomolecules on specific binding sites of a packed bed adsorbent of porous resin beads, followed by elution of the purified product. Despite the merits of this method, it still suffers from several drawbacks which might negatively affect the efficiency of the separation, mainly due to the slow intraparticle diffusion of the large biomolecules within the porous resin beads. Consequently, low operational flow rates should be maintained in order to keep the pressure limits at the accepted levels, and this will require long residence times to achieve maximum binding capacities of the target biomolecules to the binding sites on the resin. Furthermore, this method is not well suited for biomolecules with very large molecular weights (> 250 kDa) as these molecules fail to access the binding sites. Therefore, recent studies utilized nanofibers instead of the traditional chromatographic adsorbents to facilitate adsorption of biomolecules on the surface, thus eliminating intraparticle diffusion. It has been reported that separation via nanofibers can be achieved at flow rates that are ten times higher than that of packed bed chromatography, hence enabling rapid bioseparation of large biomolecules (> 250 kDa) which are difficult to separate using packed beds [83–85].

**Figure 4:**
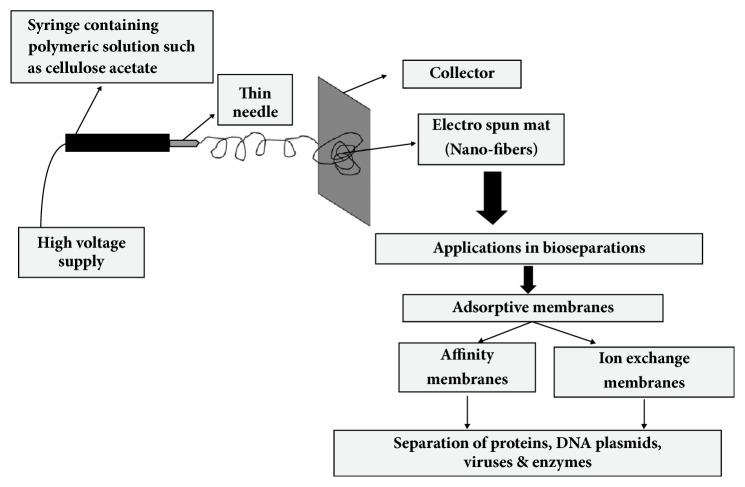
Electrospinner and the applications of nanofibers in bioseparations.

Nanofibers are produced using a technique called electrospinning where a high voltage electric current is deployed to spin many types of polymers. The polymeric solution is inserted inside a syringe which is connected to a high voltage supply that forces the solution through the thin syringe needle by overcoming the solution surface tension. The formed spun is then collected in sizes ranging from 10 – 1000 nm (Figure 4) [86–88]. Many types of natural (such as chitosan and gelatin) and synthetic (such as poly vinyl alcohol and poly vinyl pyrrolidone) polymeric solutions can be used for the formulation of nanofibers. However, cellulose acetate (CA, natural polymer) was favored because it has many advantages over other polymers including its compatibility with other biomolecules, availability and non-toxicity [89]. Nevertheless, the difficulty of finding suitable solvents for solubilizing CA remains challenging.

For bioseparation purposes, nanofibers were used in developing adsorptive affinity and ion exchange membranes which separate biomolecules based on chemical and electrostatic interactions, respectively. Selective affinity membranes adsorb biomolecules using specific ligands which are placed onto their surface. Thus, the interaction between the biomolecules and membrane depends mainly on the physicochemical properties and biological functions of the targeted molecules, rather than their molecular weights or sizes [90, 91]. Some examples were reported for the successful bioseparations of large biomolecules using adsorptive casted and nanofiber membranes such as the separation of plasmid DNA and large proteins like thyroglobulin, and small and moderate sized proteins like BSA which were successfully separated with diameters of 1- 2*μ*m [92–96].

Since the selectivity of the membrane to target biomolecules is key for separation, several attempts were made to functionalize the surface of nanofiber membranes with specific ligands that have high affinity to the target biomolecule [90, 91]. A regenerated cellulose (RC) nanofiber membrane successfully separated 13 mg/g of BSA and 4 mg/g of bilirubin. Cellulose was regenerated with alkaline medium to remove the acetyl groups and enhance its solubility and the produced membrane surface was functionalized with covalently attached cibacron blue F3GA. This membrane was characterized by its ability to be regenerated using the suitable buffer and, hence, can be reused for further separations [94]. The same workers designed a method for the purification and separation of IgG from its mixture with BSA. They again prepared regenerated cellulose nanofiber affinity membranes using electrospinning, as mentioned earlier, and functionalized them this time with protein A and protein G ligand molecules. These proteins are produced by* Staphylococcus aureus* and have affinity binding sites which strongly bind to the Fc region of IgG. Functionalization was carried out by partial oxidation of the RC nanofibers using NaIO_4_ generating aldehyde groups upon which ligand molecules can be attached covalently. This membrane was able to capture 18 *μ*g/mg IgG which is twice that achieved by the commercially affinity membrane for IgG purification (Satorbind®) [97]. In a different study, lipase enzyme (from* Candida rugosa*) was immobilized onto the surface of RC membranes by covalent bonding with surface aldehyde groups. Reaction conditions were carefully optimized at NaIO_4_ concentration of (2–10 mg/mL), reaction time (2–10 h), reaction temperature (25–35°C) and reaction pH (5.5–6.5) [98].

Electrospun CA nanofibers were also utilized as ion exchange membranes for protein separations. RC nanofibers were exposed to surface functionalization using diethylaminoethyl (DEAE) anion exchange ligands in order to separate BSA. A static binding capacity of 40 mg/g was attained as opposed to 33.5 mg/g, 14.5 mg/g, and 15.5 mg/g obtained in case of the functionalized commercial membranes: cellulose, microfiber, and cotton balls, respectively [99]. Furthermore, a higher dynamic adsorption capacity of 26.9 mg/g was obtained using DEAE nanofiber as opposed to 20.9 mg/g for DEAE commercial membrane, both measured at 10% breakthrough. In another study, the performance of nanofiber DEAE adsorbents in separating a two-component protein system (BSA and cytochrome c) using a simulated moving bed (SMB) was evaluated. The nanofiber adsorbent produced 3.92 g product/mL adsorbent/h, which is considered significant in comparison to conventional packed bed resins [100]. A summary of the above studies on nanofibers is given in Table 3.

## 5. Conclusion

Various nanoenabled processes have been recently developed to separate different biomolecules. These emerged in an attempt to address the challenges of the traditional methods of bioseparations such as centrifugation, filtration, precipitation and chromatography using conventional resins. In this regard, a number of nanoforms were used to separate target biomolecules for production purposes such as proteins, peptides, amino acids, bacteria, and enzymes. The most popular nanoforms involved in bioseparations were casted and nanofiber membranes, carbon nanotubes and magnetic nanoparticles; and they constituted polymeric/organic as well as inorganic carbon-based, silica-based, and iron oxide based materials. The latter was utilized in the form of MNPs to increase the surface area of separation, reduce agglomeration, permit the use of external magnetic field which accelerates the bioseparation process, as well as facilitate the separation of a broad range of different-sized biomolecules such as proteins and cells. However, MNPs still suffer limitations with regard to their performance due to their vulnerability to oxidation and agglomeration. In addition, achieving high adsorption capacity and selectivity is particularly challenging with non-functionalized MNPs. Functionalization was thus introduced to improve their biotargeting capacity and enhance their physicochemical properties. Functionalized MNPs were successful in separating different biomolecules such as BSA, histidine tagged, streptavidin, and trypsin proteins via hydrophobic and affinity interactions, in addition to DNA, bacteria, cells and genes via electrostatic and affinity interactions. More than 95% recoveries were achieved in most cases.

As for carbon-based inorganic materials, CNTs were favored over other spherical NPs due to their large internal volumes which allow the incorporation of different biological targets, their exceptional mechanical strengths and their unique inner and outer surfaces which facilitate their functionalization by other ligands. More specifically, MWCNTs were the most favorable due to their low cost, ease of handling, enhanced paramagnetic properties, and high binding capacity to different bacterial strains probably via hydrophobic forces without the need for functionalization. The latter property is particularly important for various food, biomedical, and environmental applications.

Recently, the application of MPMs with controlled pore sizes proved successful in the separation of complex biomolecules such as DNA, amino acids, proteins and enzymes via either electrostatic interactions or size exclusion. In this regard, polymeric-based, silica-based, and alumina oxide-based MPMs were used for the separation of binary mixtures such as BSA/BHb, lysozyme/myoglobin, and L-glutamine/L-glutamate, as well as multicomponent mixtures such as proteins in urine, and mixtures of amino acids.

With the development in electrospinning, nanofibers were introduced in the separation of large biomolecules with molecular weights above 250 kDa, and which cannot be separated by the traditional chromatographic adsorbents because they fail to access the binding sites within the porous resin beads. Adsorption onto the surface of nanofibers eliminates intra-particle diffusion and thus separation can be achieved at flow rates that are ten times higher than those of packed bed columns, hence enabling rapid bioseparation of large biomolecules. To date, very few studies were reported on the applications of nanofibers in bioseparation. Only functionalized RC nanofibers were utilized in separating BSA/bilirubin mixture, BSA, Immunoglobulin G and lipase enzyme.

## 6. Future Prospects and Challenges

In spite of the development in the design of nanoenabled processes for production bioseparations, many challenges are yet to be addressed. The state-of-the-art body of literature that focuses on such processes is limited and has still not fully tackled the shortcomings associated with them.

Nanoenabled magnetic separations showed promising potential in recovering various biomolecules with high efficiencies. However due to their limitations, there is a need for functionalization of MNPs which will unfortunately add more technical sophistication as well as additional incurred cost to the synthesis process. An additional aspect to be considered in the synthesis process is its environmental impact. Hence, the possibility of adopting green synthesis routes as alternatives to the traditional solvent intensive ones should be investigated. One emerging green process for synthesizing MNPs is based on utilizing plants and natural resources for the reduction of metal ions.

Concerning CNTs, their applications in the field of bioseparation is still very limited and mostly restricted to the non-functionalized forms. Utilizing different functionalized CNTs such as peptide CNTs and organosilica polymeric CNTs would potentially lead to improvement and versatility in its applications.

As for nanomembranes, they are notoriously known to suffer from issues of membrane fouling and concentration polarization which adversely affect its performance as a result of reduced flux, increased transmembrane pressure and, in turn, additional energy consumption requirements. In bioseparations, the possibility of membrane biodegradation by means of the biofoulants poses an additional challenge.

Despite the endeavors in developing novel nanomaterials for effective bioseparations, several nanoforms have not yet been investigated such as quantum dots, responsive polymers, dendrimers, and zeolites. For instance, dendrimers are very unique in having inner cavities and outer branched structures that facilitate holding biomolecules on their surface or entrapping them within the inner cavities. Furthermore, future studies should focus on testing the mechanical behavior of the currently used nanomaterials as well as their stability under various conditions along with their biocompatibility and toxicity in order to be suited for bioseparations.

One of the biggest challenges in the design of nanoenabled bioseparations is the scaling up of these processes. One means of scaling-up is developing column chromatography systems with functionalized nanomaterials as their stationary phase. An alternative means could be utilizing nanofiber membrane systems. However, cost effectiveness and environmental impact of these processes should be assessed. Developing more environmentally-friendly bioseparations with reduced requirements of solvents and hazardous chemicals is crucial in view of the current global economic and environmental challenges. As for highly valuable products that are demanded in relatively small amounts, the use of microfluidic devices should be explored.

Finally, nanoenabled bioseparations should target a wider spectrum of biomolecules such as insulin, whey proteins and other critical proteins, viruses and enzymes that are currently produced by the pharmaceutical companies via the conventional methods. However, scaling up could be an issue and therefore the focus should perhaps be on the products that do not require mass production.

To sum up, the utilization of nanoenabled bioseparations for production purposes is yet in its infancy stage and has not been developed on an industrial scale. It might, however, be promising in terms of efficiency, selectivity and fast operation particularly for valuable products that are not produced on a mass production scale. In that regard, lessons could be learnt from some of the success stories in the application of nanoenabled bioseparations for diagnosis and detection.

## Figures and Tables

**Table 1:** Summary of reported studies on the use of functionalized MNPs in the separation of different biomolecules.

**Targeted Biomolecules**	**Core (matrix)**	**Functionalization**	**Conditions of separation**	**Type of interaction**	**Separation Indicator**	**Reference**
**SH-SY5Y cell**	Fe_3_O_4_	PAA PEI	(i) 16 h (ii) 72 h	Electrostatic	27% PAA-MNPs 54% PEI-MNPs 58% PAA-MNPs 100% PEI-MNPs	[29]

**Lysozyme**	Fe_3_O_4_/silica Fe_3_O_4_/silica	- Polyacrylic acid	-	Electrostatic Electrostatic	127 mg/g 22 times higher	[30]

**BSA**	Silica coated MNPs	Alkyl chains	-	Hydrophobic	-	[31]

**Trypsin**	Fe_3_O_4_	Carboxylic acid groups	-	Affinity	36mg/g	[33]

**Poly Histidine tagged proteins (His-tag protein)**	Fe_3_O_4_	Nickel/silicate shell	(i) Phosphate buffer saline (PBS) (ii) 4°C	Affinity	97.28%	[34]

**His-tag protein**	Fe_2_O_3_-DA	(i) Nitrilo acetic acid (NTA)/Nickel	(i) 0.5 M NaCl (ii) 2% SDS (iii) pH 7.9	Affinity	-	[35]

**His-tagged green fluorescent protein (GFP)**	Fe_3_O_4_/PAM	NTA-Ni^2+^	Imidazole eluent (pH=8.0, 20 mM PBS, 500 mM NaCl, 250 mM and 500 mM imidazole)	Affinity	93.37%	[36]

**Streptavidin protein**	Silica NPs (2nm)	(i) Mutiple layers of Fe_3_O_4_. (ii) Extra layer of silica (iii) Bio-conjugation: Biotin	PBS	Affinity	70-90%	[37]

**Salmon sperm DNA**	Fe_3_O_4_	Mesoporous silica	(i) pH: 7.4 (ii) High salt concentration (chaotropic conditions)	Electrostatic	(i) Recovery: 100% from the surface of the nanocomposite (ii) < 10% from the magnetite core	[41]

**CD3** ^**+**^ ** T-cells from spleen**	Fe_3_O_4_	Anti-CD 3 monoclonal antibody	Phosphate buffer saline (PBS) - 4°C	Affinity	98.4 %	[48]

**Table 2:** Types of MPMs used in the bioseparation of different biomolecules.

**Biological sample**	**Type of membrane**		**Conditions of separation**	**Performance indicator**	**References**
	**Matrix**	**Functional group**			
**(BSA) and (BHb)** **∗**	poly(carbonate)	(i) Au (ii) Carboxylic acid groups	(i) pH 4.7 (ii) Ionic strength: 0.01 M	(BSA/BHb) flux ratio: 67	[74]

**(BSA) and (BHb)**	Anodic aluminum oxide (AAO)	-	(i) pH 4.7 (ii) Ionic strength: 0.01 M	(i) High selectivity: > 42 (ii) High throughput: > 10^−8^ Mcm^−2^ s^−1^	[77]

**Lysozyme/myoglobin **	Mesoporous silica (SBA-15)	-	(i) pH 3.8 (ii) pH 4.5 (iii) pH 10.6	(i) Lysozyme (pH 3.8): 6 *µ*mol/g (ii) Myoglobin (pH 4.5): 11 *µ*mol/g (iii) Lysozyme (pH 10.6): 11 *µ*mol/g	[78]

** Myoglobin**	Polymer mixture of PCGF, PEI and PLGA	-	pH 5 pH 7 pH 9	Transport rate across membrane (g m^−2^ h^−1^) 0.27 0.42 0.51	[79]

**Cytochrome C**	Polymer mixture of PCGF, PEI and PLGA	-	pH 5 pH 7 pH 9	Transport rate across membrane (g m^−2^ h^−1^) 0.38 0.58 0.54	[79]

**Proteins in urine**	polycarbonate	-	100 mM TBE buffer, pH 8.5 with 0.5% hydroxypropyl methyl cellulose (HPMC)	(i) Linear range of 0−100 *μ*g mL^−1^ (ii) LOD: 1.5 *μ*g mL^−1^, (iii) HSA recovery: 81.2−116.8%.	[80]

**Glycine, l-alanine, l-serine, l-glutamine, and l-lysine**	porous alumina	poly(styrene sulfonate)/poly(allylamine hydrochloride) (PSS/PAH)	(i) 4.8 bar (ii) 0.001 M (iii) 18 mL/min	selectivity of glycine over l-glutamine: 50 flux: 1.3m^3^/(m^2^ day)	[81]

**L-glutamine/glutamate **	Polymer	-	(i) pH 7 (ii) 16 bar	(i) > 90% rejections of glutamate and nearly 85% passage of l-glutamine. (ii) l-glutamine/glutamate selectivity: 21.7 flux: 0.81m^3^/(m^2^ day)	[82]

*∗*BSA = Bovine Serum Albumin.

*∗*BHb = Bovine Hemoglobin.

**Table 3:** A summary of the bioseparations conducted using nanofibers.

**Targeted Biomolecules**	**Membrane**	**Functionalization**	**Conditions of separation**	**Type of interaction**	**Binding capacity**	**Reference**
**BSA and bilirubin**	RC nanofiber	Cibacron Blue F3GA (CB)	For BSA: (i) PBS (pH = 7.4) (ii) Mechanical shaking under 37°C for 3 h For bilirubin: (i) Dark room (ii) PBS, (pH = 8) (iii) Mechanical shaking under 37°C for 3 h	affinity	(i) BSA: 13 mg/g (ii) Bilirubin: 4 mg/g	[94]

**BSA**	RC nanofiber	Diethylaminoethyl (DEAE) anion-exchange ligand	(i) Tris buffer (pH 8)	ion exchange electrostatic	(i) Static binding capacity: 40.0 mg/g (ii) Dynamic binding capacity at 10% breakthrough: 26.9 mg/g	[99]

**Immunoglobulin G (IgG)**	RC nanofiber	Protein A/G	1: 1 (v: v) ImmunoPure^R^ (A/G): PBS	affinity (covalent bonding)	18 *µ*g/mg	[97]

**Lipase enzyme **	RC nanofiber	Aldehyde groups	(i) 4.2mg/mL NaIO4 (ii) 6.8 h (iii) 30.8°C (iv) pH 6.1	affinity (covalent bonding)	29.6 U/g lipase activity	[98]

**BSA and cytochrome c**	RC nanofiber	Diethylaminoethyl (DEAE) anion-exchange ligand	(i) 20 mM Bis-Tris (ii) 25°C (iii) pH 5.3	ion exchange electrostatic	Static binding capacity: 10 mg/mL	[100]

## References

[1] Allhoff F., Lin P., Moore D. (2010). *What is nanotechnology and why does it matter? From science to ethics*.

[2] Pomogailo A., Kestelman V. (2015). *Metallopolymers Nanocomposites*.

[3] Wang E. C., Wang A. Z. (2014). Nanoparticles and their applications in cell and molecular biology. *Integrative Biology*.

[4] Cristofanilli M., Hayes D. F., Budd G. T. (2005). Circulating tumor cells: a novel prognostic factor for newly diagnosed metastatic breast cancer. *Journal of Clinical Oncology*.

[5] Marszałł M. P., Sroka W. D., Sikora A. (2016). Ligand fishing using new chitosan based functionalized Androgen Receptor magnetic particles. *Journal of Pharmaceutical and Biomedical Analysis*.

[6] Wubshet S. G., Brighente I. M. C., Moaddel R. (2015). Magnetic Ligand Fishing as a Targeting Tool for HPLC-HRMS-SPE-NMR: *α*-Glucosidase Inhibitory Ligands and Alkylresorcinol Glycosides from Eugenia catharinae. *Journal of Natural Products*.

[7] Safarík I., Safaríková M. (1999). Use of magnetic techniques for the isolation of cells. *Journal of Chromatography B Biomedical Sciences and Applications*.

[8] Dhadge V. L., Morgado P. I., Freitas F. (2014). An extracellular polymer at the interface of magnetic bioseparations. *Journal of the Royal Society Interface*.

[9] Jeong U., Teng X., Wang Y. (2009). Superparamagnetic colloids: controlled synthesis and niche applications. *Advanced Materials*.

[10] Nguyen D. T., Kim K. (2013). Analysis on development of magnetite hollow spheres through one-pot solvothermal process. *AIChE Journal*.

[11] Laurent S., Forge D., Port M. (2008). Magnetic iron oxide nanoparticles: synthesis, stabilization, vectorization, physicochemical characterizations, and biological applications. *Chemical Reviews*.

[12] Safarik I., Safarikova M. (2004). Magnetic techniques for the isolation and purification of proteins and peptides. *BioMagnetic Research and Technology*.

[13] Sun C., Lee J. S. H., Zhang M. (2008). Magnetic nanoparticles in MR imaging and drug delivery. *Advanced Drug Delivery Reviews*.

[14] Xu P., Zeng G. M., Huang D. L. (2012). Use of iron oxide nanomaterials in wastewater treatment: a review. *Science of the Total Environment*.

[15] Pankhurst Q. A., Connolly J., Jones S. K. (2003). Applications of magnetic nanoparticles in biomedicine. *Journal of Physics D: Applied Physics*.

[16] Tartaj P., Del Puerto Morales M., Veintemillas-Verdaguer S. (2003). The preparation of magnetic nanoparticles for applications in biomedicine. *Journal of Physics D: Applied Physics*.

[17] Lalatonne Y., Richardi J., Pileni M. P. (2004). Van der Waals versus dipolar forces controlling mesoscopic organizations of magnetic nanocrystals. *Nature Materials*.

[18] Lu A. H., Salabas E. L., Schüth F. (2007). Magnetic nanoparticles: synthesis, protection, functionalization, and application. *Angewandte Chemie International Edition*.

[19] Teja A. S., Koh P.-Y. (2009). Synthesis, properties, and applications of magnetic iron oxide nanoparticles. *Progress in Crystal Growth and Characterization of Materials*.

[20] Xu J., Sun J., Wang Y., Sheng J., Wang F., Sun M. (2014). Application of Iron Magnetic Nanoparticles in Protein Immobilization. *Molecules*.

[21] Demirer G. S., Okur A. C., Kizilel S. (2015). Synthesis and design of biologically inspired biocompatible iron oxide nanoparticles for biomedical applications. *Journal of Materials Chemistry B*.

[22] Fang W., Chen X., Zheng N. (2010). Superparamagnetic core-shell polymer particles for efficient purification of his-tagged proteins. *Journal of Materials Chemistry*.

[23] Churchill H., Teng H., Hazen R. M. (2004). Correlation of pH-dependent surface interaction forces to amino acid adsorption: Implications for the origin of life. *American Mineralogist*.

[24] Hayes M., Cuttle L., Kempf M. A method of treatment, Google Patents.

[25] Tenzer S., Docter D., Kuharev J. (2013). Rapid formation of plasma protein corona critically affects nanoparticle pathophysiology. *Nature Nanotechnology*.

[26] Lambert J. (2008). Adsorption and Polymerization of Amino Acids on Mineral Surfaces: A Review. *Origins of Life and Evolution of Biospheres*.

[27] Schwaminger S. P., García P. F., Merck G. K. (2015). Nature of interactions of amino acids with bare magnetite nanoparticles. *The Journal of Physical Chemistry C*.

[28] Burns N., Holmberg K., Brink C. (1996). Influence of surface charge on protein. *Journal of Colloid and Interface Science*.

[29] Calatayud M. P., Sanz B., Raffa V., Riggio C., Ibarra M. R., Goya G. F. (2014). The effect of surface charge of functionalized Fe_3_O_4_ nanoparticles on protein adsorption and cell uptake. *Biomaterials*.

[30] Shao D., Xu K., Song X., Hu J., Yang W., Wang C. (2009). Effective adsorption and separation of lysozyme with PAA-modified Fe3O4@silica core/shell microspheres. *Journal of Colloid and Interface Science*.

[31] Chang J. H., Lee J., Jeong Y. (2010). Hydrophobic partitioning approach to efficient protein separation with magnetic nanoparticles. *Analytical Biochemistry*.

[32] Fatima H., Kim K. (2017). Magnetic nanoparticles for bioseparation. *Korean Journal of Chemical Engineering*.

[33] Khng H. P., Cunliffe D., Davies S., Turner N. A., Vulfson E. N. (1998). The synthesis of sub-micron magnetic particles and their use for preparative purification of proteins. *Biotechnology and Bioengineering*.

[34] Liu Z., Li M., Yang X., Yin M., Ren J., Qu X. (2011). The use of multifunctional magnetic mesoporous core/shell heteronanostructures in a biomolecule separation system. *Biomaterials*.

[35] Xu C., Xu K., Gu H. (2004). Dopamine as a robust anchor to immobilize functional molecules on the iron oxide shell of magnetic nanoparticles. *Journal of the American Chemical Society*.

[36] Guo H., Li M., Tu S. (2018). Selective binding and magnetic separation of His-tagged proteins using Fe3O4/PAM/NTA-Ni2+ Magnetic Nanoparticles. *IOP Conference Series: Materials Science and Engineering*.

[37] Kyeong S., Jeong C., Kang H. (2015). Double-layer magnetic nanoparticle-embedded silica particles for efficient bio-separation. *PLoS ONE*.

[38] Woda J., Schneider B., Patel K., Mistry K., Berman H. M. (1998). An analysis of the relationship between hydration and protein-DNA interactions. *Biophysical Journal*.

[39] Weiner P. K., Langridge R., Blaney J. M., Schaefer R., Kollman P. A. (1982). Electrostatic potential molecular surfaces. *Proceedings of the National Acadamy of Sciences of the United States of America*.

[40] Pavlin M., Bregar V. B. (2012). Stability of nanoparticle suspensions in different biologically relevant media. *Digest Journal of Nanomaterials and Biostructures*.

[41] Melzak K. A., Sherwood C. S., Turner R. F. B., Haynes C. A. (1996). Driving forces for DNA adsorption to silica in perchlorate solutions. *Journal of Colloid and Interface Science*.

[42] Bruce I. J., Taylor J., Todd M., Daviesb M. J., Borionib E., Sangregorioa C. (2004). Synthesis, characterisation and application of silica-magnetite nanocomposites. *Journal of Magnetism and Magnetic Materials*.

[43] Sen T., Sebastianelli A., Bruce I. J. (2006). Mesoporous silica-magnetite nanocomposite: Fabrication and applications in magnetic bioseparations. *Journal of the American Chemical Society*.

[44] Park T. G., Jeong J. H., Kim S. W. (2006). Current status of polymeric gene delivery systems. *Advanced Drug Delivery Reviews*.

[45] Earhart C. M., Hughes C. E., Gaster R. S. (2014). Isolation and mutational analysis of circulating tumor cells from lung cancer patients with magnetic sifters and biochips. *Lab on a Chip*.

[46] Zhang L., Zhu X., Jiao D., Sun Y., Sun H. (2013). Efficient purification of His-tagged protein by superparamagnetic Fe 3O4/Au-ANTA-Co2 + nanoparticles. *Materials Science and Engineering C: Materials for Biological Applications*.

[47] Intorasoot S., Srirung R., Intorasoot A., Ngamratanapaiboon S. (2009). Application of gelatin-coated magnetic particles for isolation of genomic DNA from bacterial cells. *Analytical Biochemistry*.

[48] Cui Y.-R., Hong C., Zhou Y.-L., Li Y., Gao X.-M., Zhang X.-X. (2011). Synthesis of orientedly bioconjugated core/shell Fe 3O 4@Au magnetic nanoparticles for cell separation. *Talanta*.

[49] Baughman R. H., Zakhidov A. A., De Heer W. A. (2002). Carbon nanotubes—the route toward applications. *Science*.

[50] Mitchell D. T., Lee S. B., Trofin L. (2002). Smart nanotubes for bioseparations and biocatalysis. *Journal of the American Chemical Society*.

[51] Lee S. B., Mitchell D. T., Trofin L., Nevanen T. K., Söderlund H., Martin C. R. (2002). Antibody-based bio-nanotube membranes for enantiomeric drug separations. *Science*.

[52] Linsky J. P., Paul T. R., Kenney M. E. (1971). Planar organosilicon polymers. *Journal of Polymer Science Part A-2: Polymer Physics*.

[53] Yager P., Schoen P. E. (1984). Formation of Tubules by a Polymerizable Surfactant. *Molecular Crystals and Liquid Crystals*.

[54] Iijima S. (1991). Helical microtubules of graphitic carbon. *Nature*.

[55] Ghadiri M. R., Granja J. R., Milligan R. A., McRee D. E., Khazanovich N. (1993). Self-assembling organic nanotubes based on a cyclic peptide architecture. *Nature*.

[56] Srivastava A., Srivastava O. N., Talapatra S., Vajtai R., Ajayan P. M. (2004). Carbon nanotube filters. *Nature Materials*.

[57] Rojas-Chapana J. A., Correa-Duarte M. A., Ren Z., Kempa K., Giersig M. (2004). Enhanced introduction of gold nanoparticles into vital acidothiobacillus ferrooxidans by carbon nanotube-based microwave electroporation. *Nano Letters*.

[58] Moon H.-M., Kim J.-W. (2010). Carbon nanotube clusters as universal bacterial adsorbents and magnetic separation agents. *Biotechnology Progress*.

[59] Kim J.-W., Shashkov E. V., Galanzha E. I., Kotagiri N., Zharov V. P. (2007). Photothermal antimicrobial nanotherapy and nanodiagnostics with self-assembling carbon nanotube clusters. *Lasers in Surgery and Medicine*.

[60] Diao X., Chen H., Zhang G., Zhang F., Fan X. (2012). Magnetic Carbon Nanotubes for Protein Separation. *Journal of Nanomaterials*.

[61] Adiga S. P., Jin C., Curtiss L. A., Monteiro-Riviere N. A., Narayan R. J. (2009). Nanoporous membranes for medical and biological applications. *Wiley Interdisciplinary Reviews: Nanomedicine and Nanobiotechnology*.

[62] Felsovalyi F., Mangiagalli P., Bureau C., Kumar S. K., Banta S. (2011). Reversibility of the adsorption of lysozyme on silica. *Langmuir*.

[63] Striemer C. C., Gaborski T. R., McGrath J. L., Fauchet P. M. (2007). Charge- and size-based separation of macromolecules using ultrathin silicon membranes. *Nature*.

[64] Tong H. D., Jansen H. V., Gadgil V. J. (2004). Silicon nitride nanosieve membrane. *Nano Letters*.

[65] Létant S. E., Van Buuren T. W., Terminello L. J. (2004). Nanochannel arrays on silicon platforms by electrochemistry. *Nano Letters*.

[66] Chayen N. E., Saridakis E., Sear R. P. (2006). Experiment and theory for heterogeneous nucleation of protein crystals in a porous medium. *Proceedings of the National Acadamy of Sciences of the United States of America*.

[67] Van Meel J. A., Sear R. P., Frenkel D. (2010). Design principles for broad-spectrum protein-crystal nucleants with nanoscale pits. *Physical Review Letters*.

[68] Yang S. Y., Ryu I., Kim H. Y., Kim J. K., Jang S. K., Russell T. P. (2006). Nanoporous membranes with ultrahigh selectivity and flux for the filtration of viruses. *Advanced Materials*.

[69] Causserand C., Kara Y., Aimar P. (2001). Protein fractionation using selective adsorption on clay surface before filtration. *Journal of Membrane Science*.

[70] Sun Z., Deng Y., Wei J., Gu D., Tu B., Zhao D. (2011). Hierarchically ordered macro-/mesoporous silica monolith: Tuning macropore entrance size for size-selective adsorption of proteins. *Chemistry of Materials*.

[71] Qian K., Zhou L., Zhang J. (2014). A combo-pore approach for the programmable extraction of peptides/proteins. *Nanoscale*.

[72] Hu Y., Gopal A., Lin K. (2011). Microfluidic enrichment of small proteins from complex biological mixture on nanoporous silica chip. *Biomicrofluidics*.

[73] Fujii E., Ohkubo M., Tsuru K. (2006). Selective Protein Adsorption Property and Structure of Nano-crystalline Hydroxy-Carbonate Apatite. *Acta Biomaterialia*.

[74] Ku J.-R., Stroeve P. (2004). Protein diffusion in charged nanotubes: “On-Off” behavior of molecular transport. *Langmuir*.

[75] Bain C., Biebuyck H., Whitesides G. (1989). Comparison of self-assembled monolayers on gold: coadsorption of thiols and disulfides. *Langmuir*.

[76] Behrens S. H., Borkovec M. (1999). Electrostatic Interaction of Colloidal Surfaces with Variable Charge. *The Journal of Physical Chemistry B*.

[77] Osmanbeyoglu H. U., Hur T. B., Kim H. K. (2009). Thin alumina nanoporous membranes for similar size biomolecule separation. *Journal of Membrane Science*.

[78] Moerz S. T., Huber P. (2015). pH-Dependent Selective Protein Adsorption into Mesoporous Silica. *The Journal of Physical Chemistry C*.

[79] Schuster C., Rodler A., Tscheliessnig R., Jungbauer A. (2018). Freely suspended perforated polymer nanomembranes for protein separations. *Scientific Reports*.

[80] Hong S. U., Bruening M. L. (2006). Separation of amino acid mixtures using multilayer polyelectrolyte nanofiltration membranes. *Journal of Membrane Science*.

[81] Li S.-L., Li C., Liu Y.-S., Wang X.-L., Cao Z.-A. (2003). Separation of L-glutamine from fermentation broth by nanofiltration. *Journal of Membrane Science*.

[82] Li F., Guijt R. M., Breadmore M. C. (2016). Nanoporous Membranes for Microfluidic Concentration Prior to Electrophoretic Separation of Proteins in Urine. *Analytical Chemistry*.

[83] Levison P. (2003). Large-scale ion-exchange column chromatography of proteins Comparison of different formats. *Journal of Chromatography B*.

[84] Ghosh R. (2002). Protein separation using membrane chromatography: Opportunities and challenges. *Journal of Chromatography A*.

[85] Charcosset C. (1998). Review: Purification of proteins by membrane chromatography. *Journal of Chemical Technology and Biotechnology*.

[86] Ramakrishna S., Fujihara K., Teo W. (2005). *An Introduction to Electrospinning and Nanofibers*.

[87] Fong H., Nalwa H. S. (2007). Electrospun polymer, ceramic, carbon/graphite nanofibers and their applications. *Polymeric Nanostructures and Their Applications*.

[88] Reneker D. H., Chun I. (1996). Nanometre diameter fibres of polymer, produced by electrospinning. *Nanotechnology*.

[89] Kulpinski P. (2005). Cellulose nanofibers prepared by the N-methylmorpholine-N-oxide method. *Journal of Applied Polymer Science*.

[90] Thömmes J., Kula M. R. (1995). Membrane Chromatography—An Integrative Concept in the Downstream Processing of Proteins. *Biotechnology Progress*.

[91] Klein E. (2000). Affinity membranes: A 10-year review. *Journal of Membrane Science*.

[92] Teeters M. A., Conrardy S. E., Thomas B. L., Root T. W., Lightfoot E. N. (2003). Adsorptive membrane chromatography for purification of plasmid DNA. *Journal of Chromatography A*.

[93] Yang H., Viera C., Fischer J., Etzel M. R. (2002). Purification of a large protein using ion exchange Membranes. *Industrial & Engineering Chemistry Research*.

[94] Ma Z., Kotaki M., Ramakrishna S. (2005). Electrospun cellulose nanofiber as affinity membrane. *Journal of Membrane Science*.

[95] Ma Z.-W., Kotaki M., Ramakrishna S. (2006). Surface modified nonwoven polysulphone (PSU) fiber mesh by electrospinning: a novel affinity membrane. *Journal of Membrane Science*.

[96] Bamford C., Al-Lamee K., Purbrick M., Wear T. (1992). Studies of a novel membrane for affinity separations. *Journal of Chromatography A*.

[97] Ma Z., Ramakrishna S. (2008). Electrospun regenerated cellulose nanofiber affinity membrane functionalized with protein A/G for IgG purification. *Journal of Membrane Science*.

[98] Huang X.-J., Chen P.-C., Huang F., Ou Y., Chen M.-R., Xu Z.-K. (2011). Immobilization of *Candida rugosa* lipase on electrospun cellulose nanofiber membrane. *Journal of Molecular Catalysis B: Enzymatic*.

[99] Zhang L., Menkhaus T. J., Fong H. (2008). Fabrication and bioseparation studies of adsorptive membranes/felts made from electrospun cellulose acetate nanofibers. *Journal of Membrane Science*.

[100] Hardick O., Dods S., Stevens B., Bracewell D. G. (2015). Nanofiber adsorbents for high productivity continuous downstream processing. *Journal of Biotechnology*.

